# α2,3-Sialyltransferase ST3Gal III Modulates Pancreatic Cancer Cell Motility and Adhesion *In Vitro* and Enhances Its Metastatic Potential *In Vivo*

**DOI:** 10.1371/journal.pone.0012524

**Published:** 2010-09-01

**Authors:** Marta Pérez-Garay, Beatriz Arteta, Lluís Pagès, Rafael de Llorens, Carme de Bolòs, Fernando Vidal-Vanaclocha, Rosa Peracaula

**Affiliations:** 1 Biochemistry and Molecular Biology Unit, Department of Biology, University of Girona, Girona, Spain; 2 Department of Cell Biology and Histology, School of Medicine and Dentistry, Basque Country University, Leioa, Spain; 3 Cancer Research Program, IMIM-Hospital del Mar, Barcelona, Spain; University of Hong Kong, Hong Kong

## Abstract

**Background:**

Cell surface sialylation is emerging as an important feature of cancer cell metastasis. Sialyltransferase expression has been reported to be altered in tumours and may account for the formation of sialylated tumour antigens. We have focused on the influence of alpha-2,3-sialyltransferase ST3Gal III in key steps of the pancreatic tumorigenic process.

**Methodology/Principal Findings:**

ST3Gal III overexpressing pancreatic adenocarcinoma cell lines Capan-1 and MDAPanc-28 were generated. They showed an increase of the tumour associated antigen sialyl-Lewis^x^. The transfectants' E-selectin binding capacity was proportional to cell surface sialyl-Lewis^x^ levels. Cellular migration positively correlated with ST3Gal III and sialyl-Lewis^x^ levels. Moreover, intrasplenic injection of the ST3Gal III transfected cells into athymic nude mice showed a decrease in survival and higher metastasis formation when compared to the mock cells.

**Conclusion:**

In summary, the overexpression of ST3Gal III in these pancreatic adenocarcinoma cell lines underlines the role of this enzyme and its product in key steps of tumour progression such as adhesion, migration and metastasis formation.

## Introduction

Cell surface sialylation is emerging as an important feature of cancer cell metastasis. Sialic acids and their derivatives are ubiquitous at terminal positions of glycoconjugates. Those acidic sugars impart net negative charge and are in a position to modulate a wide variety of events in cell-cell, cell-matrix and cell-molecule interactions [1]. The transfer of the sialic acids from cystidine-5-monophospho-N-acetylneuraminic acid (CMP-NeuAc) to the terminal positions of carbohydrate groups of glycoprotein and glycolipids is catalyzed by sialyltransferases [2].

Human sialyltransferases are a family of 20 different intracellular, Golgi membrane-bound glycosyltransferases; grouped in three subfamilies [3]. Alpha-2,6-sialyltransferases mediate the transfer of sialic acid with an alpha 2,6-linkage to terminal Gal (ST6Gal I-II) [4], [5]or GalNAc residues (ST6Gal NAc I-VI). Alpha-2,8-sialyltransferases mediate the transfer of sialic acid with an alpha 2,8-linkage (ST8 Sia I-IV). Alpha-2,3-sialyltransferases mediate the transfer of sialic acid with an alpha 2,3-linkage to terminal Gal residues. ST3Gal I-II and IV catalyze the transference to the Gal residue located on terminal Galβ1-3GalNAc structures; ST3Gal IV and VI transfer sialic acid (with alpha 2,3-linkage) to the Gal residue located on terminal Galβ1-4GlcNAc structures; ST3Gal V acts on the Gal residue located on terminal Galβ1-4Glc-Cer structures and finally, ST3Gal III catalyzes the transfer of sialic acid with an alpha 2,3-linkage to terminal Gal residues located on either Galβ1-3GlcNAc or Galβ1-4GlcNAc structures [6].

Changes in specific sialyltransferase expression have been reported to be altered in several tumours and may account for the formation of sialylated tumour antigens, such as sialyl-Lewis x, sialyl- Lewis a, sialyl-T and sialyl- Tn. In the extrahepatic bile duct carcinoma ST3Gal III levels correlated with tumour advancement, differentiation and metastasis [7]. In breast cancer, the most expressed sialyltransferase was ST3Gal III which positively correlated to tumour size and the number of axilary nodes; and moreover high ST3Gal III/ST6Gal I ratio was correlated with a shorter overall survival and bad prognosis [8], [9]. In addition, ST6GalNAc V has recently been reported to mediate brain metastasis of breast cancer cells [10]. In bladder cancer ST3Gal I plays the major role in the sialylation of the T antigen and its overexpression seems to be part of the initial oncogenic transformation [11]. In cervix squamous cell carcinoma, ST6Gal I and ST3Gal III expression levels were significantly increased in patients with lymph node metastasis when compared to those without metastases [12], [13] and ST3Gal III, ST3Gal IV and ST6Gal I were increased in cervical intraepithelial neoplasia. In human renal carcinoma a down-regulation of ST3Gal IV mRNA may be one of the factors associated with its malignant progression [14]. In colon cancer ST6Gal I and ST3Gal III increased their expression in carcinoma specimens [15]. ST3Gal III was prominently increased in cancer tissues compared with non-malignant colorectal mucosa [16] and an elevation of ST6Gal I activity was observed in malignant and transitional tissue [17]. In gastric cancer, high levels of ST3Gal III a in the tumour tissue correlated with secondary tumour recurrence [18].

Although alpha-2,3-sialyltransferase ST3Gal III expression correlates with tumour malignancy in several carcinomas its mechanistic role has not been fully evaluated. ST3Gal III is involved in the biosynthesis of sialyl-Lewis antigens, which are overexpressed in pancreatic adenocarcinoma (PDAC) [19], [20], [21], [22], [23], [24], and correlate with its bad prognosis [25], [26], [27], [28]. In the present work, our goal has been to investigate the specific influence of the alpha-2,3-sialyltransferase ST3Gal III in some of the key steps of the PDAC progression such as adhesion, migration and metastasis. For that, we have chosen two pancreatic adenocarcinoma cancer cell lines Capan-1 and MDAPanc-28, with different ST3Gal III and sialyl-Lewis antigen expression levels, and generated ST3Gal III overexpressing clones. ST3Gal III increased expression was related to an increase sialyl-Lewis^x^ surface level and conducted to an enhanced E-Selectin binding capacity and cell migration. Furthermore, the intrasplenic injection into athymic nude mice of the ST3Gal III overepressing cells showed a decrease in mice survival and higher metastasis formation. In summary, increased expression of ST3Gal III in those pancreatic adenocarcinoma cell lines highlights the role of this enzyme and its product in key steps of tumour progression such as adhesion, migration and metastasis.

## Results

### Stable overexpression of ST3Gal III in Capan-1 and MDAPanc-28 cells

**Figure 1:**
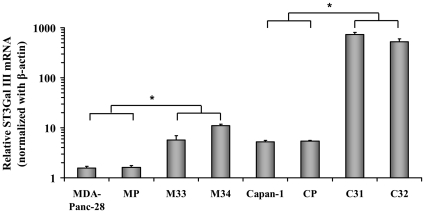
ST3Gal III expression normalized to β-actin of the pancreatic adenocarcionoma cells. MDAPanc-28 parental cells, MP: MDAPanc-28 mock cells, M34 and M33: MDAPanc-28 cells transfected with the ST3Gal III gene. Capan-1**:** parental cells, CP: Capan-1 mock cells, C31 and C32: Capan-1 cells transfected with the ST3Gal III gene. Data represents the mean ± SD of 3 separate experiments, each in six replicates (n = 18). * Significantly different (*P<0.001*).

To explore the mechanistic role of ST3Gal III in pancreatic adenocarcinoma progression, the rat ST3Gal III gene, which exhibits virtually identical acceptor specificity and enzymatic activity as human ST3Gal III gene [29], was used. Thus, Capan-1 and MDAPanc-28 cells were transfected with the pcDNA 3.1 vector encoding rat ST3Gal III gene. Parental cells were concomitantly transfected with the empty pcDNA3.1 vector. Several stable cell clones were selected in the presence of geneticine and ST3Gal III mRNA overexpression was analysed by semi-quantitative PCR (data not shown). ST3Gal III mRNA overexpressing clones, C31 and C32 (for the Capan-1 model) and M33 and M34 (for the MDAPanc-28 model) were selected for further studies. As controls, parental cell lines (Capan-1 and MDAPanc-28) and mock transfected clones (CP for Capan-1 and MP for MDA-Panc 28) were used. ST3Gal III mRNA expression was quantified by quantitative PCR (qPCR) (**Figure 1**). Capan-1 parental cells had 3-fold higher (*P<0.001*) ST3Gal III expression than MDAPanc-28 parental cells. Regarding the Capan-1 model, ST3Gal III mRNA expression was 140-fold higher (*P<0.001*) in C31 clone and 100-fold higher (P<0.001) in C32 clone than in Capan-1 and CP control cells. For the MDAPanc-28 model, ST3Gal III mRNA expression was 7-fold higher (*P<0.001*) in M34 clone and 3.6-fold higher (P<0.001) in M33 clone than in both control cells, MDAPanc-28 and MP. Evaluation of general sialyltransferase activity was performed by Glycosylation Immunosorbent assay (GISA) as previously described [24]. As expected, a general sialyltransferase activity increase was detected in the transfected cells when compared to their corresponding controls, being about 3.2 fold higher in the Capan-1 ST3Gal III overexpressing clones *versus* CP and Capan-1 and 1.3 fold higher in the MDAPanc-28 ST3Gal III overexpressing clones *versus* MP and MDAPanc-28.

### ST3Gal III increased de novo expression of SLex by enzymatic competition

**Figure 2:**
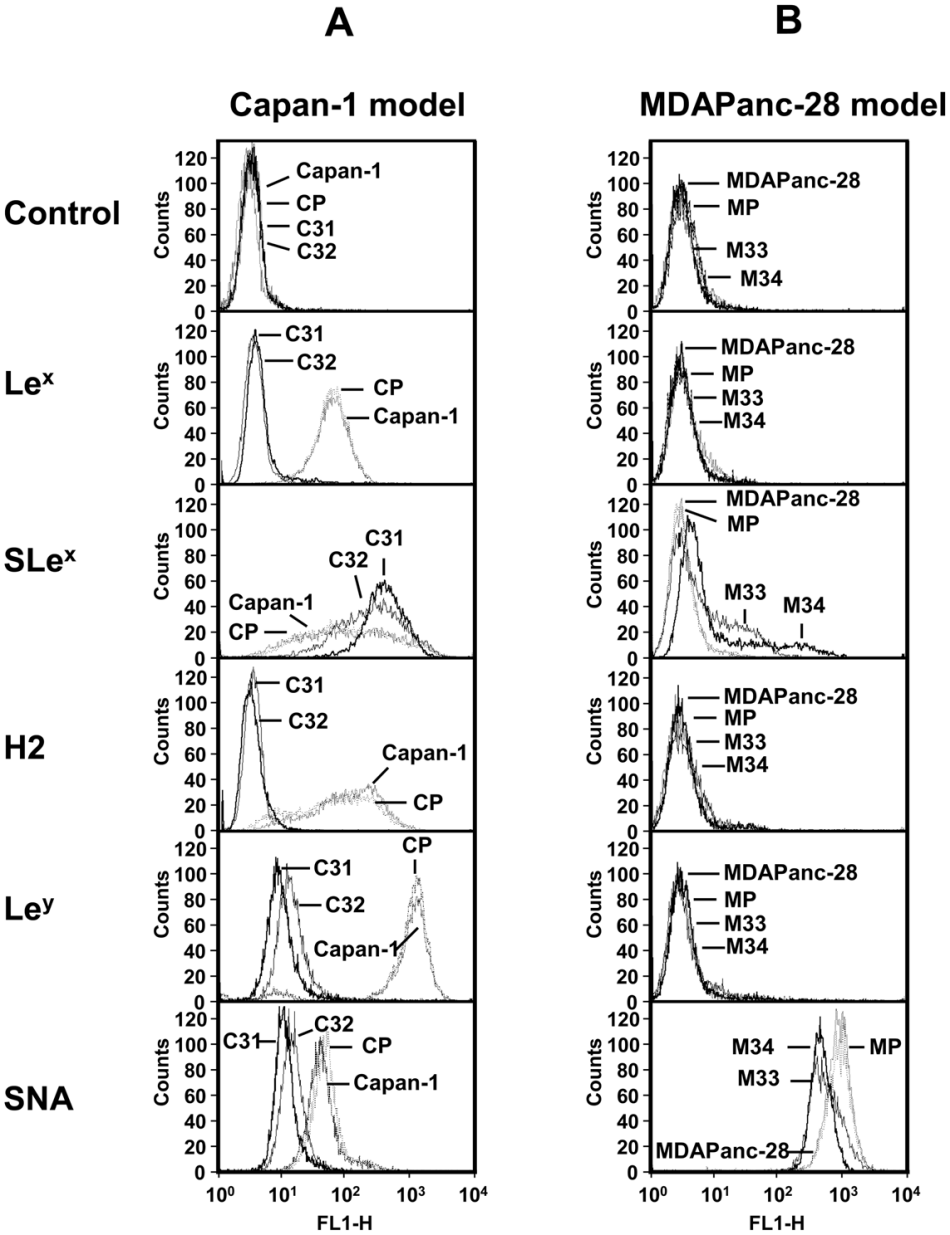
Flow cytometry profiles of the cell surface glycan structures of the pancreatic adenocarcionoma cells. **Figure 2A**. Capan-1 (continous dot outline: ………), CP (spaced dot outline: . . . . . ), C31 (bold outline:_______), C32 (plain outline:______). **Figure 2B**. MDAPanc-28 (continous dot outline: ………), MP (spaced dot outline: . . . . . ), M34 (bold outline:_______), M33 (plain outline:______). Experiments were performed for triplicate. Representative cytometry histograms are shown. Anti-Le^x^ MAb binds to Galβ1,4[Fucα1,3]GlcNAc-; anti-SLe^x^ MAb binds to NeuAcα2-3Galβ1,4[Fucα1,3]GlcNAc-; anti-H2 MAb binds to [Fucα1,2]Galβ1,4GlcNAc-; anti-Le^y^ MAb binds to [Fucα1,2]Galβ1,4[Fucα1,3]GlcNAc-; SNA lectin (*Sambucus nigra* agglutinin) binds to NeuAcα2–6Galβ- structures.

Cell surface glycan expression pattern for both models was studied by flow cytometry using specific monoclonal antibodies against Lewis antigens and specific lectins (results shown in **Figure 2**). The analysis of Type II Lewis antigens in the Capan-1 model (**Figure 2A**) revealed that Capan-1 and CP cells had high-medium levels of the Le^x^, SLe^x^ and H2 antigens and high levels of the Le^y^ antigen. When compared to controls, C31 and C32 clones displayed a large increase in SLe^x^ expression at the expense of completely losing the expression of non-sialylated antigens (Le^x^, H2 and Le^y^). Moreover, accompanying the increase in SLe^x^, a decrease in α2,6-sialic acid was observed, detected by *Sambucus nigra agglutinin* (SNA). *Maackia amurensis agglutinin* (MAA) did not show differences among clones, probably due to the fact that is unable to bind to SLe^x^ structure (data not shown). Type I Lewis antigens SLe^a^, Le^a^, Le^b^ and H1 were not detected in the Capan-1 model (data not shown). Thus, these results evidenced a multiple enzymatic competition for Type II chains. The analysis of MDAPanc-28 (**Figure 2B**) model showed that MDAPanc-28 and MP control cells had very low SLe^x^ expression and high α2,6-sialic acid expression and also lacked Type I Lewis antigens. When M33 and M34 clones were compared to controls, an increase in SLe^x^ with a simultaneous decrease in α2,6-sialic acid was also detected. As above, MAA did not show differences between clones likely because it does not recognize SLe^x^ (data not shown). Since ST3Gal III transfected clones C31 and C32 behaved similarly in the Capan-1 model as well as the M33 and M34 ST3Gal III transfected clones in the MDAPanc-28 model, the following *in vitro* and *in vivo* studies were performed only with the highest ST3Gal III and SLe^x^ expressing clones: C31 for the Capan-1 model and M34 for the MDAPanc-28 model.

### ST3Gal III enhanced pancreatic adenocarcinoma cells adhesion to rh-E-selectin

**Figure 3:**
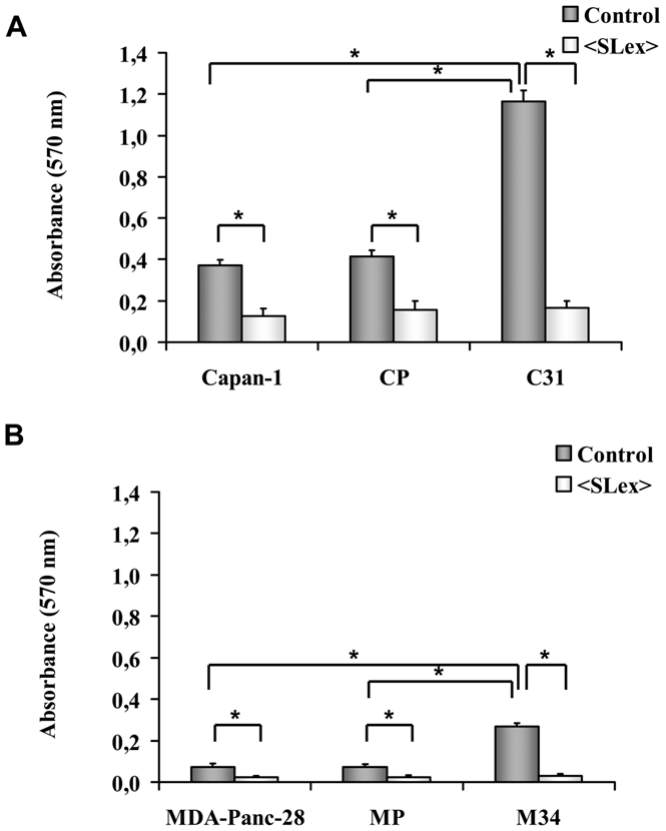
Binding assay to rh-E-selectin. Capan-1 variant cells (**Figure 3A**) and MDAPanc-28 variant cells (**Figure 3B**), previously incubated with PBS-1% BSA (light bars) or anti-SLe^x^ MAb (dark bars), were added to 96-well microplates coated with rh E-selectin or PBS-1% BSA (negative control). Adherent cells were estimated with a MTT-based colorimetric assay. Results are expressed as the Specific binding to E-selectin (O.D. 570 nm of cells bounded to E-selectin – O.D. 570 nm of cells bonded to PBS-1% BSA) *versus* cells previously incubated or not with anti-SLe^x^ MAb. Data represents the mean ± SD of 3 separate experiments, each in five replicates (n = 15). * Significantly different (*P<0.001*).

E-selectin is a cell adhesion molecule expressed on activated endothelial cells that recognizes and binds to specific carbohydrate determinants, such as SLe^x^ and SLe^a^ present on surface glycoconjugates [30]. Binding assays to rh-E-selectin were performed to analyse whether the different pattern of the Lewis antigen expression was able to induce changes in the adhesion (results shown in **Figure 3**). Both models showed different adhesion patterns to rh-E-selectin. MDAPanc-28 parental cells displayed lower adhesion to rh-E-selectin than Capan-1 parental cells, which was consistent with the lower expression level of ST3Gal III and SLe^x^ of MDAPanc-28 compared to Capan-1 cells. In the Capan-1 model (**Figure 3A**) the ST3Gal III overexpressing clone, C31, tripled the adhesion (*P<0.001*) to rh-E-selectin when compared to corresponding controls Capan-1 and CP. To confirm that SLe^x^ expression induced by ST3Gal III caused the up-regulated E-selectin binding, cells were previously incubated with the anti-SLe^x^ monoclonal antibody (MAb) and the binding to E-selectin was inhibited. In the MDAPanc-28 model (**Figure 3B**), the ST3Gal III overexpressing clone, M34, quadrupled (*P<0.001*) the adhesion to rh-E-selectin when compared to the corresponding controls MDAPanc-28 and MP. When cells were previously incubated with the anti- SLe^x^ MAb the binding to E-selectin was completely abrogated. These results demonstrated that, in these models, ST3Gal III levels modulated the *in vitro* binding to rh-E-selectin via SLe^x^ expression.

### The effect of different cytokines on the adhesion of pancreatic cancer cells to hepatic sinusoidal endothelial (HSE) cells

**Figure 4:**
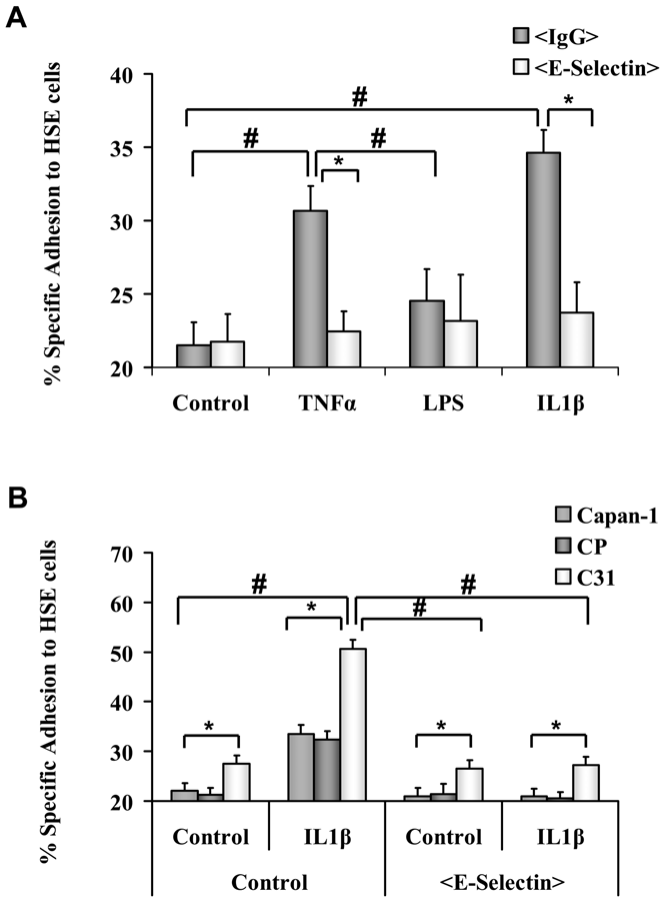
E-selectin induction on Primary Cultured Hepatic Sinusoidalendothelium (HSE) cells (Figure 4A). HSE cells were incubated with <Sel>  =  anti-murine CD62 E (E-selectin) MAb or <IgG>  =  isotype-matched control antibody. Parental Capan-1 cells were labelled with calcein and added to HSE control cells, TNF-α stimulated HSE cells, LPS stimulated HSE cells or IL-1β stimulated HSE cells. Results are expressed as the % Specific adhesion to HSE cells [78]. Data represents the mean ± SD of 3 separate experiments, each in three replicates (n = 9). * Significantly different (*P<0.001*) when comparing anti-E-selectin incubated HSEC to control cells. # Significantly different (*P<0.001*) when comparing treatments. **Tumour cell adhesion assay to Primary Cultured HSE cells (** **Figure 4B** **).** Capan-1 variant cells were labelled with Calcein and added to IL-1β stimulated HSE cells or (**−**) not stimulated HSE control cells. <Sel**>**  =  anti-murine CD62 E (E-selectin) MAb was added to IL-1β HSE cells or **-** HSE cells before tumour cell addition. * Significantly different (*P<0.001*) when comparing Capan-1, CP, and C31 cells. # Significantly different (*P<0.001*) when comparing each clone (Capan-1, CP and C31) adhesion for the different HSE cell treatments.

Because ST3Gal III and SLe^x^ expression levels were directly proportional to *in vitro* rh-E-selectin adhesion, the binding of pancreatic cancer cells to primary cultured HSE cells was evaluated. The Capan-1 model was selected for this experiment due its higher adhesion to rh-E-selectin. First, we determined the specific adhesion of Capan-1 parental cells to HSEC stimulated with TNF-α, IL1-β or lipopolysaccharide (LPS) for 16 hours before the assay (results shown in **Figure 4A**). Treatment with TNF-α and IL1-β greatly increased the number of adherent cells, and IL1-β resulted to be the best stimuli for our model. When incubating with anti-E-selectin MAb, the increase in adhesion was completely abrogated. Those data showed that HSE cell cytokine pre-treatment, especially IL-1β, promoted their E-selectin expression, which caused an increase of Capan-1 adhesion.

### ST3Gal III increased tumour cell adhesion to HSE cells

We next determined the adhesion of different ST3Gal III and SLe^x^ levels expressing Capan-1 cells to IL-1β pre-treated or control HSE cells (results shown in **Figure 4B**). Capan-1 and CP control cells showed a basal adhesion to HSE cells, which significantly increased in IL-1β pre-treated HSE cells and returned to basal levels in anti-E-selectin MAb pre-incubated HSE cells. C31 showed a basal adhesion to HSE cells (higher than Capan-1 and CP control cells), which significantly increased in IL-1β pre-treated HSE cells and also returned to basal levels in anti-E-selectin MAb preincubated HSE cells. Those data showed that E-selectin played an important role mediating adhesion to HSE cells, although a non-E-selectin dependent basal adhesion (higher for C31 than for control cells) existed. Moreover, high ST3Gal III and SLe^x^ expressing C31 cells had a higher adhesion to HSE cells than medium ST3Gal III and SLe^x^ expressing control cells.

### ST3Gal III expression increased cell migration

**Figure 5:**
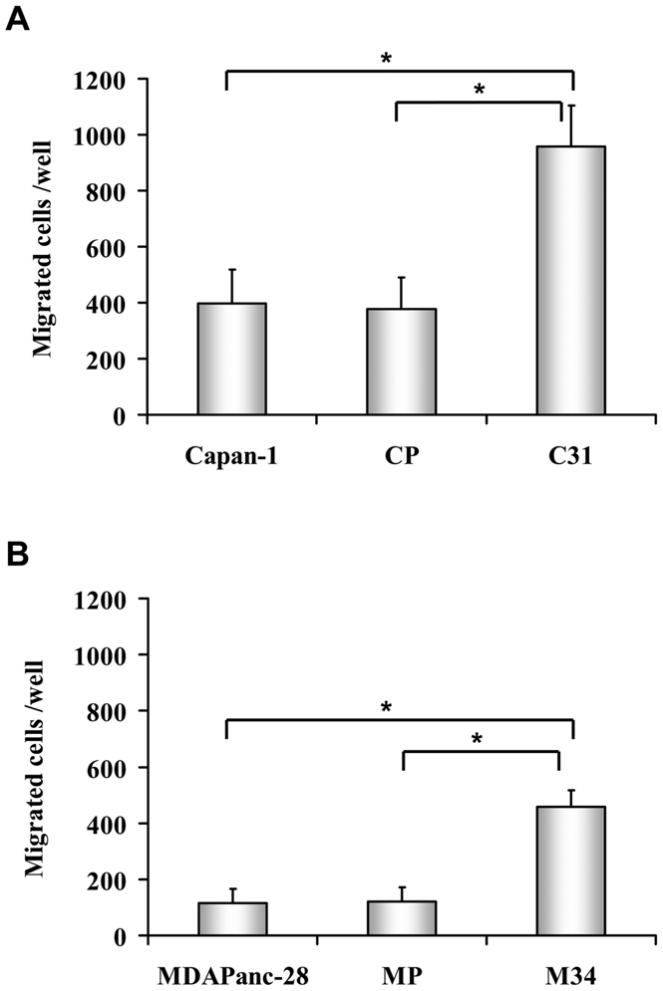
Cell migration assay. Capan-1 variant cells (Capan-1,CP and C31) *(* *Figure 5A* *)* and MDAPanc-28 variant cells (MDAPanc-28, MP and M34) *(* *Figure 5B* *)* were seeded onto 8 µm-pores-Type I-Collagen coated inserts, placed on top of wells containing DMEM-1% FBS and incubated at 37°C (6 h for Capan-1 model and 18 h for MDAPanc-28 model). Non-migrated cells were eliminated and migrated cells fixed, stained and counted. Results are expressed as migrated cells per well. Data represents the mean ± SD of the values obtained in 3 separate experiments, (n = 9). * Significantly different (*P<0.001*).

To investigate whether ST3Gal III overexpressing clones might lead to the acquisition of a more migratory cell phenotype, we evaluated cell migration on collagen Type-I using a transwell migration assay (results shown in **Figure 5**). Both cell models showed different migratory capabilities, with the Capan-1 model being nine times more migratory than the MDAPanc-28 model. Regarding the Capan-1 model (**Figure 5A**), the ST3 Gal III and SLe^x^ overexpressing clone C31 exhibited double (*P<0.001*) migration capabilities than control cells Capan-1 and CP. For the MDAPanc-28 model (**Figure 5B**), the ST3Gal III and SLe^x^ overexpressing clone M34 increased migration by four with respect to the control cells MDAPanc-28 and MP. Results demonstrated a positive correlation between ST3Gal III and SLe^x^ levels and migratory capabilities.

### ST3Gal III overexpression increases tumourigenesis and decreases survival in athymic nude mice

**Figure 6:**
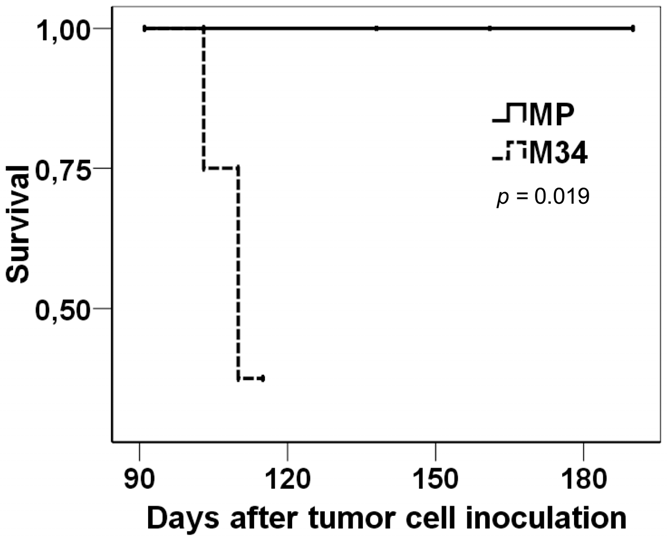
Kaplan-Meier plots of estimated survival after injection of MP (MDAPanc-28 mock cells) and M34 (MDAPanc-28 ST3Gal III transfected cells). Cells (7×10^6^) were intrasplenically injected in nude mice on day 1 of the experiment. Mice were daily examined and sacrificed when they looked sick. The differences between groups were assessed by the long-rank test (*P* = 0.019; n = 7–8/group).

Since the *in vitro* results described above suggested an important role for the ST3Gal III gene in tumour progression, *in vivo* assays were performed to study whether ST3Gal III could be important in tumour metastasis. First, the formation of metastasis and survival of athymic nude mice after intrasplenic injection with different amounts of the Capan-1 and MDAPanc-28 parental cells were assayed. In our hands, when 1.5×10^6^, 3×10^6^ and 5×10^6^ Capan-1 cells were injected, the nude mice died of emboli formation, probably due to the Capan-1 size and aggregation capacity. The injection of 1×10^6^ Capan-1 cells did not form emboli and generated spleen tumours, although without metastatic focuses. Since MDAPanc-28 cells are 4–5 times smaller than Capan-1 cells, 7×10^6^ MDAPanc-28 cells were injected intrasplenically into nude mice, which generated small macroscopic metastatic focuses in 2/3 of the mice, 20–22 weeks later. Therefore, MP and M34 cells were chosen to study the influence of ST3Gal III overexpression in metastasis formation and nude mice survival. Exponentially growing 7×10^6^ viable MP and M34 cells, which share the same morphological characteristics, were injected in the spleen of athymic nude mice (n = 8/group) and survival analysis was performed using the Kaplan-Meier method (**Figure 6**). Mice injected with ST3Gal III overexpressing cells (M34) showed a large decrease in survival (*P = 0.019*) when compared with mice injected with control MP cells. Most of the M34 injected mice (5/8) died after 103–110 days post-injection, while MP injected mice survived until the end of the study (190 days). All M34 injected mice were necropsied and the analysis showed spleen tumours in 62.5% of them (5/8) and several macroscopic metastatic focuses to the bowel, diaphragm, kidney, ganglia or suprarenal glands in 75% of the mice (6/8). All MP injected mice were necropsied (days 103, 138, 161 and 190 post-injection) and none of them (8/8) showed evidence of either spleen tumours or macroscopic metastasis. Thus, the overexpression of ST3Gal III in MDAPanc-28 cells, which lead to a higher expression of SLe^x^ and a higher adhesion and migration, could be directly correlated with a decrease in survival when injected in nude mice. Furthermore, it endowed cells with a greater tumour formation capability and metastatic potential when intrasplenically injected.

## Discussion

Our earlier studies showed that alpha-2,3-sialyltransferase activity correlated to sialyl-Lewis antigen expression on pancreatic cancer cell surfaces [24]. We therein extended our investigations to specifically study the mechanistic role of ST3Gal III in the acquisition of adhesive, migratory and metastatic capabilities in pancreatic adenocarcinoma cancer cell lines. We have found that: 1) ST3Gal III mRNA expression levels correlated with SLe^x^ surface expression; 2) ST3Gal III induced sialylation increased pancreatic cancer adhesion to rh-E-Selectin and IL1-β pre-stimulated HSE cells, via SLe^x^-E-selectin interaction; 3) a positive correlation between migration and ST3Gal III and SLe^x^ expression; and 4) the intrasplenic injection of ST3Gal III overexpressing cell lines in athymic nude mice induced an increase in tumourigenesis and a decrease in survival.

ST3Gal III overexpressing clones (C31, C32, M33 and M34) displayed an increase in SLe^x^ expression when compared to the corresponding controls (untransfected cells, Capan-1 and MDAPanc-28, and cells transfected with the empty vector, CP and MP). Although Type I substrate is considered ST3Gal III preferred acceptor, Type II substrate is also a good acceptor. The enzymatic studies carried out in order to characterize ST3Gal III activity showed that Type I and Type II acceptor substrates led to quite similar V_max_ values, while K_m_ values were much higher for type II. As a consequence, incorporation rates were approximately twice for Type I than for Type II structures [31]. Similar results were obtained in later works on studying natural [32], [33] or synthetic acceptors specificities [34], [35], [36], [37], [38]. Taking into account that the cell lines used in this study do not express type I Lewis antigens and that ST3Gal III can act on both type I and II substrates, the overexpression of ST3Gal III acted on type II precursors leading to an increased expression of SLe^x^. Recently, and in agreement with our results, it has been described that overexpression of ST3Gal III in gastrointestinal carcinoma cells also resulted in SLe^x^ determinant increase [39]. Moreover, these authors described that the mRNA levels of ST3Gal III correlated with increased SLe^x^ expression levels in confluent cells, but not with the mRNA levels of ST3Gal IV and ST3Gal VI, which are the enzymes that have been reported to act preferentially on Type II chains. Taken together, these data reinforce the role of ST3Gal III in the biosynthesis of SLe^x^ in these carcinoma cells. Together with SLe^x^ increase, a decrease in α2,6-sialic acid was observed in both models. In addition, C31 and C32 showed a complete loss of non-sialylated Type II Lewis antigens (Le^x^, H2 and Le^y^). These results are likely explained by multiple enzymatic competition for Type II chains. On one hand, there was competition among alpha-1,2-fucosyltransferases, alpha-1,3(4) fucosyltransferases and alpha-2,3-sialyltransferases [23], [40], [41], and on the other between alpha-2,3-sialyltransferases and alpha-2,6-sialyltransferases [6], [31], [42].

The molecular mechanisms regulating pancreatic tumour metastasis are still poorly understood. One crucial step is the attachment of tumour cells to activated endothelial cells, which involves their adhesion to endothelial selectins that recognize oligosaccharide determinants expressed on cancer cells surface. SLe^x^ has been reported to participate in the first steps of the extravasation through interaction with E-Selectin by facilitating the rolling and attachment of tumour cells to the endothelial cells [27], [28], [43], [44], [45]. A direct correlation between ST3Gal III levels, SLe^x^ levels and colon cancer cell adhesion to IL-1β activated human umbilical vein endothelial cells (HUVEC) has been described [46]. SLe^x^ expressing colon cancer cells have been also reported to adhere to hepatic sinusoidal endothelial cells via E-selectin-SLe^x^ interaction [47]. Furthermore, several works reported a correlation between SLe^x^, and the adhesion to cytokine stimulated HUVEC in H7721 hepatocarcinoma [48], [49], [50], [51] and in lung adenocarcinoma cells [52]. In agreement with these data, our results showed a direct correlation between pancreatic adenocarcinoma adhesion to *in vitro* rh-E-selectin and ST3Gal III levels in pancreatic tumour cells, via SLe^x^. Moreover, when studying the adhesion of pancreatic adenocarcinoma cells to hepatic sinusoidal endothelial cells, ST3Gal III and SLe^x^ overexpressing C31 cells demonstrated an enhanced ability to adhere to IL-1β stimulated HSE cells compared with controls. HSE cell pre-treatment with IL1-β and TNF-α cytokines significantly increased Capan-1 cell adhesion to HSE cells and returned to basal levels in anti-E-selectin pre-incubated HSE cells. Those results are consistent with previous work reporting that E-selectin expression is low or absent in HSE cells under normal conditions, but can be up-regulated by cytokines [53], by hepatocytes [54] or as a response to metastatic tumour cells [55]. Adhesion of ST3Gal III and SLe^x^ overexpressing C31 cells to non-stimulated HSE cells was significantly greater when compared to control cells Capan-1 and CP. This adhesion was not reverted when pre-incubating HSE cells with anti-E-selectin, which could be explained by a non-E-selectin dependent, but SLe^x^-mediated, pancreatic cancer cell adhesion to non-stimulated HSE cells. This interaction could be mediated by other SLe^x^ binding Cell Adhesion Molecules such as P-selectin [55], [56] or I-type Lectins. In fact, a previous study reported the binding of ST3Gal III overexpressing colon cancer cells to non-activated HUVEC, suggesting this non-E-selectin dependent SLe^x^ mediated adhesion could be due to I-Type lectins [46].

To our knowledge, this is the first report on a positive correlation between cell migration and ST3Gal III and SLe^x^ expression levels in pancreatic adenocarcinoma cells. Despite the absence of studies on pancreatic cancer cells, some investigations support our findings. When tumour cell surface α2,3-sialic acid expression on the MDA-MB-231 breast cancer cell line was depressed (by inhibiting cellular α2,3-sialyltransferase activity with soyasaponin I), cell migration significantly decreased [57]. ST3Gal III overexpression on the glioma cell line U-374 increased α2,3-linked sialic acid expression on cell surface and resulted in a more *in vitro* invasive phenotype [58]. In addition, H7721 hepatocarcinoma cells showed a direct correlation between the amount of surface SLe^x^ expression and migration [50], [51], [59]. Moreover, SLe^x^ was crucial for H7721 migration, since migration was inhibited by anti-SLe^x^ MAb and after sialic acid residue elimination [60]. Cell migration is a multistep process that plays a pivotal role in metastasis. The initial response of migratory cells to a migration-promoting agent is to polarize and extend protrusions. These protrusions are stabilized by extracellular matrix adhesion molecules, such as integrins, and serve as traction sites for migration as the cell moves forward over them [61]. There is a rapidly growing body of evidence that demonstrates that sialylation influences migration capabilities by modulating the integrin function [58], [62], [63], [64]. Thus, we hypothesize that ST3Gal III could be influencing cell migration by altering integrin sialylation. An increase in α2,3-sialic acid on C31 and M34 cell surface could also alter the sialylation levels of their integrins, modulating their extracellular matrix adhesion. Nevertheless, further investigations are required to address this issue.

Several studies have described a correlation between SLe^x^ levels and metastasis or poor survival in patients for several types of cancer, such as colon cancer [25], gastric carcinoma [26], breast cancer [65] and pancreatic cancer [66]. Along with those clinical observations, the expression levels of SLe^x^ on colon cancer cell surface [25], [67], [68] and gastric cancer cell surface [69] correlated with metastatic potential in animal models. Focusing on pancreatic cancer, the SLe^x^ antigen has an important *in vivo* role, since an inhibitory effect in tumour establishment and metastatic colonies growth was observed when mice were treated with anti-SLe^x^ antibody [70]. SLe^x^ cell surface levels have also been found to be important in determining the degree of metastasis formation. [71], [72] have described how excessive expression of SLe^x^ in tumour cells leads to rejection by natural killer cells rather than tumour formation, while moderate amounts of SLe^x^ lead to tumour metastasis.

In our hands, a low number of Capan-1 cells with high-medium levels of SLe^x^ did not generate metastasis when intrasplenically injected into athymic nude mice. In contrast, MDAPanc-28 cells with low levels of SLe^x^ were able to form metastases in several organs, when injected at high numbers. However, the different behaviour of these two cell lines in tumour metastasis generation could be explained by a combination of multiple factors, including gene expression pattern, which is not the aim of this work. ST3Gal III overexpression in MDAPanc-28 pancreatic cancer cells (M34 cells), which leads to medium expression of SLe^x^ levels, increased the ability to tumour establishment, metastasis growth and decreased survival when compared to MDAPanc-28 mock cells (MP cells). Thus, a correlation between ST3Gal III expression in MDAPanc-28 pancreatic cancer cells and metastasis in athymic nude mice could be established. Although this is the first study that shows a direct implication of ST3Gal III in tumour and metastasis formation, other studies had pointed in this direction. Thus, the overexpression of FUT1, which competes for the same substrate as ST3Gal III and IV, showed a decrease in the metastatic potential of pancreatic adenocarcinoma [40] and colon cancer [41] cells after injection into nude mice.

Our studies have started from the generation of stable transfectants of ST3Gal III in two different pancreatic adenocarcinoma cell lines to globally study the role of this enzyme in key steps of the tumourigenic process. In this way, we have shown that this enzyme and its main product, SLe^x^, confer on pancreatic carcinoma cells a major E-selectin adhesion, a high migration capacity and a high metastatic potential. In addition to the role of ST3Gal III in these key steps of tumourigenic processes, this enzyme have recently been reported to be involved in producing cellular resistance to Taxol in ovarian cancer cells [73]; all together highlighting the importance of ST3Gal III in tumor processes. Although ST3Gal III plays an important role in the above processes, other related sialyltransferases may also contribute to them. A next step would be to understand the regulation of these enzymes and to design inhibitors that may down-regulate their biosynthesis and inhibit these processes.

## Materials and Methods

### Ethics statement

Animal housing, their care, and experimental conditions were conducted in conformity with institutional guidelines that are in compliance with the relevant national and international laws and policies. All procedures working with animals were performed according to protocols approved by the University Ethics Commission on Research and Education of the Basque Country University, which approved this study (reference 09341).

### Stable transfection

The construct rat α2,3-sialyltransferase (rat ST3Gal III) [29] cloned into pcDNA3.1(+) was a kind gift from Dr. Paulson (The Scripps Research Institute, La Jolla, CA). The nucleotide sequence and correct orientation were confirmed by DNA sequencing (ABI prism 310 genetic analyser, Applied Biosystems, CA).The ST3Gal III encoding expression vector and the empty pcDNA 3.1 encoding expression vector were transfected into human pancreatic adenocarcinoma cell lines Capan-1 (ATCC n°HTB-79, Rockville, MD) and MDAPanc-28 (a generous gift from Dr. Frazier from M.D. Anderson Cancer Center, Houston) using Lipofectamine 2000 and Plus Reagent (Invitrogen Life Technologies, Frederick, MD). Cells in which the plasmid was stably integrated were selected with 400 µg/mL (Capan-1 cloned cells) or 800 µg/mL (MDAPanc-28 cloned cells) of Geneticine® G418 (Gibco, Paisley, UK), and resistant clones were further confirmed by semi-quantitative PCR.

### Culture conditions for transfected cells

Cells were grown in Dulbecco's modified Eagle's medium_GlutaMAX-I containing 10% Fetal Bovine Serum, 100 U/mL Penicillin G, 100 µg/mL Streptomycin and 0.25 µg/mL Amphotericin B (all of them from Gibco, Paisley, UK) and kept at 37°C in humidified atmosphere containing 5% CO_2_. Stable transfectants were supplemented with Geneticine® G-418. Cell growth and morphology were daily assessed under the field microscope. Cells were routinely maintained for up to 8 passages by successive trypsinization and seeding and cell viability was assessed by trypan blue staining (only cultures displaying >90% viability were used for further work). For the experiments 3.5×10^5^ Capan-1 model cells or 5.5×10^5^ MDAPanc-28 model cells were seeded in 75 cm^2^ flasks (Nunc; Roskilde, Denmark) and cultured during 84 h (exponential growth). Possible contamination with *Mycoplasma* was routinely checked using the Venor® GeM *Mycoplasma* Dection Kit (Minerva Biolabs GmnH, Berlin, Germany).

### Isolation of Total RNA and semi-quantitative RT-PCR for ST3 Gal III

Exponentially growing cells were trypsinized and counted. Total RNA was extracted using the RNeasy® RNA isolation kit (Quiagen, Hilden, Germany) according to the manufacturer's protocol, including on-column DNase digestion using the RNAse-Free DNase Set (Quiagen GmbH, Hilden, Germany). RNA yield and purity were determinate spectrophotometrically using a Nanodrop (ND-1000, Thermo Scientific, Wilmington, DE). Single-stranded cDNA was synthesized from 2.0 µg of total RNA using the High-Capacity cDNA Reverse Transcription Kit (Applied Biosystems Inc, Foster City, CA) according to the manufacturer's instructions.

ST3Gal III overexpression was examined by semi-quantitative PCR using the β-actin expression as internal reference. PCR was performed using 1.3*µ*L of cDNA, 2 mM MgCl_2_, 50 µM each dNTP, 18 *p*mol each forward (F) and reverse (R) oligonucleotides, and 1 unit *µ*L^–1^ Biotools DNA polymerase (Biotools B&M Labs, Madrid, Spain) in 20*µ*L reactions. ST3Gal III primers were designed with Primer3 software [74] to specifically amplify the rat ST3Gal III cloned gene. Primer sequences were 5′-CTGCATGGCTGTGATGAAGT-3′_(F) and 5′-CAACAGATGGCTGGCAACTA-3′_ (R). PCR product size was 272 bp and thermal cycling parameters were as follows: 1 cycle at 90°Cfor 2 min, 35 cycles at 94°C for 1 min, 58°C for 1 min, 72°C for 1 min and 30 s, 1 cycle at 72°C for 10 min; using a MyCycler™ thermocycler (Bio-Rad Labs Hercules, CA). β-actin primers and thermal cycling conditions were described in Peracaula et al., 2005 and the size of the PCR product obtained was 838 bp. 15 µL aliquots of amplified cDNAs were run in 1.5% agarose gels, stained with 0.5*µ*g/ml ethidium bromide, and visualized under UV-light. The intensity of the amplified cDNA bands of ST3Gal III was measured using the *Quantity-One* software package (Bio-Rad Labs Hercules, CA) and was normalized to the housekeeping gene (β-actin) for each cell line. Data were expressed as the mean ± SD of the intensity values of the PCR assays performed in triplicate.

### ST3Gal III expression by Real-time quantitative PCR

RNA extraction and cDNA synthesis were performed as described above. Primers and probes sequences for the endogen gene (β-actin) and ST3Gal III were Custom Taqman Gene Expression Assays™ from Applied Biosystems-Applera Hispania SA, Spain. ST3Gal III primers and probe were specifically designed along highly conserved regions of both rat and human ST3Gal III transcripts (positions 318 or 330). All PCRs were performed in optical 96-well plates with an ABI PRISM 7300 Sequence Detector System in a total volume of 20 µl containing 9 µl of cDNA diluted in RNAse free water, 10 µl of TaqMan® Universal Master Mix No AmpErase® and 1 µl of the corresponding Custom Taqman Gene Expression Assay™. The following standard thermal profile was used for all PCRs: 95°C for 10 min; 40 cycles of 95°C for 15 s and 60°C for 1 min. All data were processed and analyzed with 7300 SDS 1.3.1 software (Applied Biosystems). For each sample dilution, logarithmic increase in fluorescence signal (Δ*R*n) was obtained. ΔRn threshold was set at 0.02 to obtain the corresponding Ct (threshold cycle) values. The relative concentrations of the target and the reference gene were calculated by interpolation from the corresponding standard curves. The endogenous housekeeping gene β-actin was used to normalize the results. To estimate the intra-assay variability, six technical replicates were performed for each sample and gene. To estimate the inter-assay variation, PCR assays were performed in triplicate. Results were expressed as mean ± SD values of the relative ST3Gal III transcript abundance normalized with corresponding β-actin.

### GISA (Glycosylation ImmunoSorbent Assay)

GISA was performed as described previously [24]. Briefly,10 ng of asialofetuin (Roche) were bound for 1 hour at 37°C to 96-well polystyrene plates in coating buffer (Scil Diagnostics, Germany). After washing them, plates were blocked with 1% BSA, 0.05% (v/v) Tween and were incubated for two hours at 37°C with the different quantities of the cell protein extracts from Capan-1, CP, C31, MDAPanc-28, MP and M34 (5–25 µg) and 0.77 mg/L of CMP-5-fluoresceinyl -NeuAc (CMP-5-(N-fluoresceinylthioureido-acetyl-neuraminic acid) in 45 mM NaCl, 15 mM Tris-HCl, pH 7.2, 1% Triton X-100 and 0.1% BSA. Plates were washed and an antibody against FITC (fluorescein isothiocyanate), peroxidase-conjugated 1∶2000 in PBS, 0.05% Tween, 0.25% BSA was added and allowed to stand for one hour at 37°C. Plates were washed again and detection was performed with 100 µl/well of 3,3′,5,5′-tetramethylbenzidine (TMB) (BM BluePOD substrate soluble, Roche). The reaction was stopped with 100 µl/well of 0.25M H_2_SO_4_ and the absorption was measured at 450 nm (against a reference wavelength of 630 nm) in an automated microplate reader (BIO-TEK, USA). Negative controls were wells without protein extract, or without the CMP-5-fluoresceinyl-NeuAc. Positive controls were wells with recombinant rat α2,6-N-Sialyltransferase (Calbiochem, Germany) instead of the cell protein extracts.

### Antibodies and Flow Cytometry analysis

Monoclonal antibodies (MAb) T-218 (anti-Lewis b) and T174 (anti-Lewis a) [75] were used at 1∶2 dilution hybridoma supernatant; 19-OLE (anti -H type 2) and anti-H type1 [76] were used as ascites diluted at 1/1000. MAb KM93 (anti-SLe^x^), MAb P12 (anti-Le^x^), MAb KM231 (anti- SLe^a^) and MAb F3 (anti-Le^y^) (all of them from Calbiochem, EMD Chemicals, Inc. San Diego, CA). Biotinylated *Sambucus Nigra* (Elderberry Bark) Lectin, which recognizes most Siaα2,6-terminal structures and biotinylated *Maackia amurensis* lectin, which binds to certain terminal Siaα2,3-terminal structures but not to SLe^x^ determinants (Vector Laboratories, Inc. Burlingame, CA) were diluted following manufacturer's instructions. Detection of oligosaccharide epitopes on the surface of exponential growing cells was carried out by indirect fluorescence as previously described [41]. Briefly, 5×10^5^ viable cells were incubated with the antibodies or the lectin and after a wash; cells were then incubated with the secondary antibody Alexa Fluor 488 goat anti-mouse IgG or Streptavidin Alexa Fluor 488 (Invitrogen Life Technologies, Frederick, MD). Fluorescent analysis was performed using a FACSCalibur (BD Biosciences). For each sample three independent assays were undertaken.

### E-selectin binding assay

Adhesion of pancreatic adenocarcinoma cells to recombinant human E-selectin (rh-E-selectin) was performed as previously described [41]. 96-well microplates were coated with rh-E-selectin (R & D Systems, Minneapolis, USA) or 1% BSA. Plates were blocked and 5×10^4^ viable Capan-1, CP or C31 cells or 1×10^5^ MDAPanc-28, MP or M34 cells, were added and incubated at room temperature for 1 h. In selected experiments, cells were previously incubated with specific antibodies for 30 min at 4°C. After two washes, adherent cells were estimated with a Thiazolyl Blue (Sigma-Aldrich, St. Louis, MO) based colorimetric method. All the experiments were performed in quintuplicate, and three independent assays were undertaken. Results were expressed as the mean ± SD values of specific binding to E-selectin (O.D. 570 nm of cells bonded to E-selectin – O.D. 570 nm of cells bonded to PBS-1% BSA).

### Isolation and primary culture of hepatic sinusoidal endothelium (HSE) cells

Syngenic Balb/c mice (male, 6–8 weeks old) were obtained from Harlan Iberica (Barcelona, Spain). HSE cells were separated from these mice, identified, and cultured as previously described [77].

### Tumour cell adhesion assay to Primary Cultured Hepatic Sinusoidal endothelium (HSE) cells

Adhesion assays were performed using a quantitative method based on a previously described fluorescence measurement system [78]. Isolated HSE cells were incubated for 16 hours with 10 ng/ml recombinant Interleukin IL-1β, 10 ng/ml recombinant TNF-α or 10 ng/ml Lipopolysaccharide (LPS) (all from R & D Systems) before addition of cancer cells. 2 µg/mL anti-murine CD62 E (E-selectin) MAb (Acris Antibodies GmbH, Herford, Germany) was added to HSE 30 minutes before tumour cell addition. Anti-murine IgG antibody was added at a similar concentration and time to check the specificity of the anti-murine E-selectin antibody. Exponentially growing Capan-1 clones were trypsinized and resuspended in 20 µg Calcein AM (Invitrogen Life Technologies, Frederick, MD) DMEM solution. After washing, cells were resuspended in HEPES-buffered Dubelco's modified Eagle medium without phenol red at a concentration of 2×10^6^ cells per millilitre. HSE cells were washed and basal autofluorescence was determined using a CytoFluor-2350 system (Millipore Co., Bedford, MA). Labelled Capan-1 clones (0.1 mL per well) were added to 24-well-plate cultured HSE cells or to collagen pre-coated control wells. To determine the fluorescence of the added number of cells in each well, a second determination was performed on the CytoFluor system. The plates were then incubated at 37°C, and 15 minutes later, wells were washed three times with fresh medium and read for a third time for fluorescence. Each experiment was performed in triplicate wells and three independents assays were undertaken. The number of adhering cells was quantified in arbitrary fluorescence units based on the percentage of the initial number of cancer cells added to the endothelia and results were expressed as the mean ± SD values of % Specific adhesion to HSE cells [78].

### Transwell in vitro migration assays

The effect of ST3Gal III expression on Capan-1 and MDAPanc-28 cell migration was determined using modified Boyden chambers as previously described [79]. Cells were detached and resuspended in serum-free medium. 1×10^4^ Capan-1 cells or 2.5×10^4^ MDAPanc-28 cells were seeded onto Type I-Collagen coated inserts with 8 µm-pores and placed on top of 2 cm^2^ wells (Greiner Bio-One GmbH, Kremsmünster, Austria) containing 300 µL DMEM plus 1% FBS (as a chemoattractant). After incubation at 37°C, 6 hours for Capan-1 cells and 18 hours for MDAPanc-28, non-migrated cells on the upper surface of the filter were carefully and thoroughly wiped from the top surface of the filter and migrated cells were fixed, stained with Haematoxylin and Eosin (H&E) and counted in ×40 high-power light microscopy. Results were expressed as the average number of migrated cells per well obtained from three separate experiments done in triplicate.

### In vivo assays in athymic nude mice

Athymic Nude-Fox n1 nu/nu mice (male, 6–8 weeks old) weighing 21.9–24.8 g were obtained from Charles River (Barcelona, Spain). *In vivo* assay optimal conditions were assessed by intrasplenic injection into anesthetized mice (50 mg/kg pentobarbital i.p) of exponentially growing 1×10^6^, 1,5×10^6^, 3×10^6^ and 5×10^6^ Capan-1 viable cells and 7×10^6^ MDAPanc-28 viable cells suspended in 0.1 ml Hanks'Balanced solution (Gibco, UK). MDAPanc-28 model analysis was performed by intrasplenic injection into anesthetised mice (n = 8 per group) of exponentially growing 7×10^6^ MP and M34 viable cells. Mice were daily examined for survival and sacrificed when looked sick. Healthy mice from both experimental groups were also sacrificed to check for internal signs of malignancy and censored in the survival analysis. For necropsied animals, macroscopical analysis was performed and the incidence of tumour lesions in liver, spleen lung, lymph nodes, and other peritoneal organs were registered.

### Statistical Analysis

Data (x) were expressed as means ± standard deviation (SD). Statistical analyses were performed using SigmaStat 3.5 for Windows (Systat Sofware, Inc., San José, CA) and SPSS statistical software for Windows (version 15.0; SPSS Inc., Chicago, IL). Normality of data (x) was tested using the Kolmogorov-Smirnov test and the homogeneity of variances was checked using the Levene's test. Data with normal distribution and homogeneous variances were analyzed with Student's t test, one-way or three-way ANOVA using Tukey's test for multiple comparisons. For heterocedastic data, ANOVA on ranks (Kruskal-Wallis test) was run using Dunn's method for pairwise multiple comparisons. On the other hand, time to survival data in experimental metastasis assay were analyzed by Kaplan-Meier method and compared by the long-rank test. The criterion for significance was set at *P<0.05*.
